# Detecting central hypovolemia in simulated hypovolemic shock by automated feature extraction with principal component analysis

**DOI:** 10.14814/phy2.13895

**Published:** 2018-11-22

**Authors:** Björn J. P. van der Ster, Berend E. Westerhof, Wim J. Stok, Johannes J. van Lieshout

**Affiliations:** ^1^ Department of Internal Medicine Academic Medical Center University of Amsterdam Amsterdam the Netherlands; ^2^ Department of Medical Biology Academic Medical Center University of Amsterdam Amsterdam the Netherlands; ^3^ Laboratory for Clinical Cardiovascular Physiology Center for Heart Failure Research Academic Medical Center Amsterdam the Netherlands; ^4^ Department of Pulmonary Diseases Amsterdam Cardiovascular Sciences VU University Medical Center Amsterdam the Netherlands; ^5^ MRC/Arthritis Research UK Centre for Musculoskeletal Ageing Research School of Life Sciences the Medical School University of Nottingham Medical School Queen's Medical Centre Nottingham United Kingdom

**Keywords:** hypovolemia, LBNP, machine learning, shock

## Abstract

Assessment of the volume status by blood pressure (BP) monitoring is difficult, since baroreflex control of BP makes it insensitive to blood loss up to about one liter. We hypothesized that a machine learning model recognizes the progression of central hypovolemia toward presyncope by extracting information of the noninvasive blood pressure waveform parametrized through principal component analysis. This was tested in healthy volunteers exposed to simulated hemorrhage by lower body negative pressure (LBNP).

Fifty‐six healthy volunteers were subjected to progressive central hypovolemia. A support vector machine was trained on the blood pressure waveform. Three classes of progressive stages of hypovolemia were defined. The model was optimized for the number of principal components and regularization parameter for penalizing misclassification (cost): C. Model performance was expressed as accuracy, mean squared error (MSE), and kappa statistic (inter‐rater agreement).

Forty‐six subjects developed presyncope of which 41 showed an increase in model classification severity from baseline to presyncope. In five of the remaining nine subjects (1 was excluded) it stagnated. Classification of samples during baseline and end‐stage LBNP had the highest accuracy (95% and 50%, respectively). Baseline and first stage of LBNP demonstrated the lowest MSE (0.01 respectively 0.32). Model MSE and accuracy did not improve for C values exceeding 0.01. Adding more than five principal components did not further improve accuracy or MSE. Increment in kappa halted after 10 principal components had been added. Automated feature extraction of the blood pressure waveform allows modeling of progressive hypovolemia with a support vector machine. The model distinguishes classes between baseline and presyncope.

## Introduction

The effective circulating blood volume refers to the part of the volume within the arterial system effectively perfusing the tissues (Schrier 1990; Abraham and Schrier 1994) and is assumed to depend mainly on the central blood volume (CBV), but clinical assessment of the CBV continues to be difficult (Schrier 1990; Marik et al. 2011; Bronzwaer et al. 2015; Secher and van Lieshout 2016).

As an example, during anesthesia volume treatment is generally planned according to a somewhat arbitrary fixed volume regime or guided by blood pressure (BP) and heart rate (HR), focusing on maintaining fluid balance. In contrast, in patients with end‐stage kidney disease being treated with hemodialysis or hemodiafiltration intravascular volume depletion is planned. With progression of the dialysis session, the preload of the heart declines and eventually may become too low to maintain a sufficient cardiac output with the development of arterial hypotension when the limits of vasomotor reserve available for vasoconstriction have been reached (Schondorf and Wieling 2000; Fu et al. 2004; Schiller et al. 2017).

Volume treatment essentially has to balance the danger of death in response to a serious reduction of CBV, the volume of blood directly available to the left ventricle, against that of developing pulmonary and/or peripheral edema (Secher and van Lieshout 2005; Godfrey et al. 2014). Considering the negative impact of either inadequate or overaggressive fluid therapy proper assessment of the volume status would benefit patient care (Kalantari et al. 2013). Therefore, early detection of a critical reduction of the CBV within the effective treatment window would be a valuable feat. Hypovolemic shock is characterized by a critically reduced CBV but loss of ~1 L of blood or fluid is regularly not reflected in blood pressure. This makes assessment of the circulatory state complex (McMichael 1944; Wiggers 1950; Sander‐Jensen et al. 1986; Harms et al. 2003; Marik et al. 2009; Zhang et al. 2012).

The integral response of the cardiovascular system to progressing central hypovolemia from rest to the stage of hemodynamic instability and presyncope was quantified earlier with the use of either a support vector machine or a neural network trained on features assumed having a physiological and/or clinical meaning (Bennis et al. 2017; van der Ster et al. 2018). By design, the majority of these features – including blood pressure and transcranial Doppler determined cerebral blood flow velocity derivatives – are the result of considerable down‐sampling of potentially sensitive features of arterial pressure and cerebral blood flow velocity waveforms. However, both the arterial pressure and transcranial cerebral blood flow velocity waveforms contain subtle information on the cardio‐cerebrovascular condition (van der Ster et al. 2018). Principal component analysis (PCA) is a mathematical technique to identify a reduced number of uncorrelated features from a larger dataset. It expresses the variation in the data in a set of orthogonal (uncorrelated) vectors. We hypothesized that by automated parametrization through PCA (Pearson 1901) these nuances enclosed within the BP waveform can be expressed numerically and thus improve precision in quantifying the cardiovascular state. This study tested in healthy volunteers subjected to progressive reduction of the CBV by lower body negative pressure (LBNP) whether principal component analysis describes the progress toward critical central hypovolemia.

## Methods

The study protocol was approved by the Academic Medical Centre Amsterdam medical ethical committee (Study no. #2014_310) and conform the standards set by the Declaration of Helsinki. Written informed consent was obtained from all subjects.

## Subjects

Fifty‐six healthy, nonsmoking volunteers (25 males) who were physically active participated in the study (age: 24, standard deviation (SD) 4 years; height: 176, SD 10 cm; weight: 71, SD 11 kg). Exclusion criteria were a medical history of cardio‐ and/or cerebrovascular disease, neurological disorders, diabetes mellitus, regular fainting, and the use of medication. Prior to the experiment subjects abstained from heavy exercise, alcohol, and caffeinated beverages for at least 12 h.

Continuous beat‐to‐beat BP was measured noninvasively using finger plethysmography (Nexfin, Edwards Lifesciences, Irvine, CA) (Martina et al. 2012). An appropriately sized finger cuff was applied to the mid‐phalanx of the middle finger of the left hand. The hand was maintained at heart level during for the duration of the measurement.

### Protocol

Lower body negative pressure (LBNP) was applied to simulate hemorrhage. Responses to LBNP and blood loss up to 1000 mL follow a similar hemodynamic stimulus‐response pattern (Johnson et al. 2014; Rickards et al. 2015). The lower part of the body was positioned inside a lower body negative pressure (LBNP) box (Kaiser Medizintechnik, Bad Hersfeld, Germany) and sealed at the level of the iliac crest (Goswami et al. 2009; Bronzwaer et al. 2017). The LBNP box was equipped with a saddle to prevent leg muscle pump activation during the application of the subatmospheric pressure. Following 30 min of supine rest, continuous negative pressure (50 mmHg below atmospheric pressure) was applied to the lower body. The pressure inside the box was manually controlled and established within 20 sec.

The protocol continued until the subject developed presyncope or tolerated LBNP for 30 min without developing presyncopal symptoms. In compliance with our laboratory safety guidelines presyncopal symptoms include sweating, light‐headedness, nausea, blurred vision, and/or signs meeting one or more of the following criteria: systolic arterial pressure (SAP) below 80 mmHg, or rapid drop in BP (SAP by ≥25 mmHg/min, diastolic arterial pressure (DAP) by ≥15 mmHg/min), and drop in HR by ≥15 bpm/min. The subjects were continuously monitored by an investigator experienced in human studies and unoccupied by experimental obligations (van der Ster et al. 2018). To account for the uncertainty in the subset of subjects that did not develop presyncope, these subjects were not included in the training set. Instead their data served as a check for false positive classification of hypovolemia.

### Preprocessing and feature extraction

**Figure 1:**
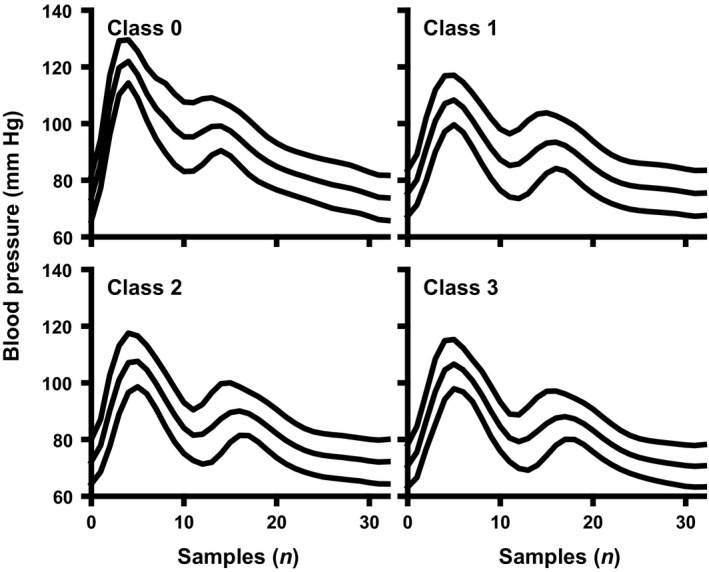
Individual blood pressure waveform. Mean and 95% confidence interval of the blood pressure waveform in a single subject for the four stages during the protocol: rest (class 0) and three stages of LBNP induced progressive hypovolemia (classes 1 through 3).

All BP waveforms were extracted from the continuous 200 Hz sampled tracing. Each wave was interpolated (*1‐dimensional cubic (Cubic Hermite splines) interpolation*) to contain exactly 33 samples and subsequently labeled with a class number (Fig. 1). Classes were defined as 0 (supine baseline rest, i.e., normovolemia), and 1 through 3 (representing 0–33%, 34–67%, and 68–100% of the segment of LBNP, respectively). Samples containing Nexfin Physiocal pressure calibrations were removed.

### Modeling

The total feature matrix consisted of ~100.000 blood pressure waves with 33 features. This matrix was converted to principal components as a way of parametrizing the blood pressure waveform which then served as input to a supervised support vector machine algorithm (libsvm software package for Matlab [Chang and Lin 2011]). This machine learning approach bases future decisions on previously measured example data samples. We used a linear kernel and tested for different regularization parameter values (C: C = 0.001; C = 0.01; C = 0.1; C = 1; C = 10; and C = 100. The parameter C is a regularization coefficient controlling the trade‐off between minimizing training errors and controlling model complexity (Bishop 2006). A lower value for C allows for a larger margin for the decision boundary (hyperplane), potentially increasing misclassification whereas a larger value for C attempts to minimize misclassification by finding a smaller margin hyperplane. The optimal value for C is used for a stepwise addition of principal components to retrieve the minimally required number of components for tackling this learning task.

The model was trained using a leave one subject out strategy (Shao et al. 2016). To limit computational time a 10‐fold bootstrap approach was applied to select a random subsample of 10% of the remaining training set data each fold. Subsequently, these 10 acquired models were tested on the integral set of the held‐out data. The resulting classification represented by discrete values between 0 and 3 was then smoothed using a moving average over 20 beats and averaged for the 10 models after bootstrapping. Model performance was expressed in terms of accuracy, kappa statistic (Cohen 1960) from the confusion matrix and mean squared error (MSE) from the proposed classes. Kappa can be used for sensitivity and specificity for more than one class and compares observed accuracy (by the model) versus expected accuracy (random chance). Thus kappa describes how close the model matches the predefined class labels while correcting for a model that would randomly classify. Overall the measure is a value that describes the performance of the model over all classes as a single number. A perfect model would have a kappa of 0.81‐1 and a poor one <0.20 (Landis and Koch 1977).

## Results

Nine out of 56 subjects completed the full 30 min of LBNP without developing presyncope and were excluded from the training set. Data of one subject was excluded because too few samples (<1 min of data) were available from the segment during LBNP, leaving 72.500 blood pressure waves originating from 46 subjects available for training and testing the model.

**Figure 2:**
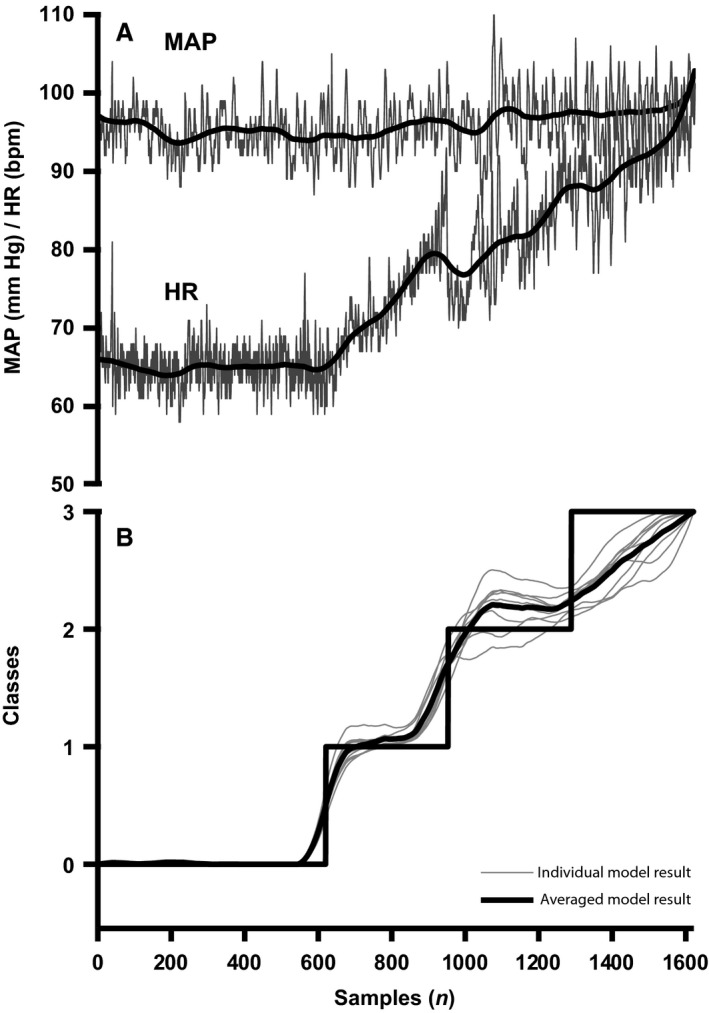
Individual hemodynamic and model responses. (A) Mean arterial pressure (MAP) and heart rate (HR) and their moving averages (bold lines). (B) 4 defined classes: rest (0) and LBNP (1 through 3) (black, stepwise line) with advancing simulated hemorrhage and the model responses following 20 sample moving averaging for a model with regularization value C = 1 for 10 bootstraps (gray) and their mean (black) for one subject.

**Figure 3:**
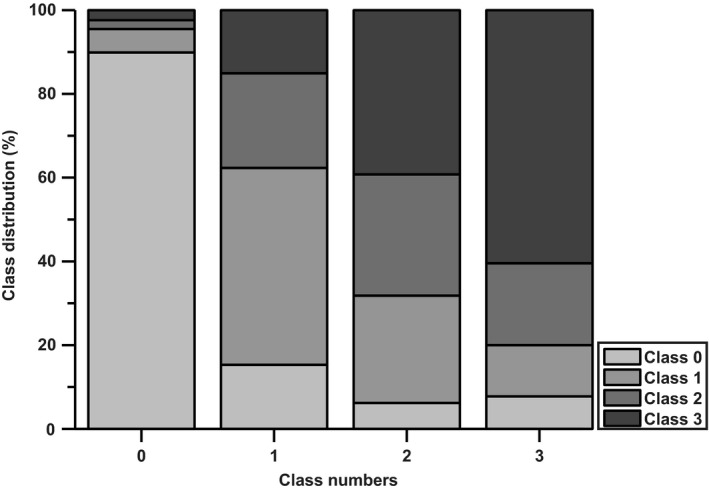
Class distribution for each class in percentages. Average classification distribution for all subjects for the different classes. Each gray shade represents a class. A perfect model should match the color of the respective class 100% of the times. Note the increase in prediction of class 3 (dark gray) during progress of hypovolemia and the decrease of class 0 (lightest gray) after the onset of LBNP (bars with classes 1 through 3).

**Figure 4:**
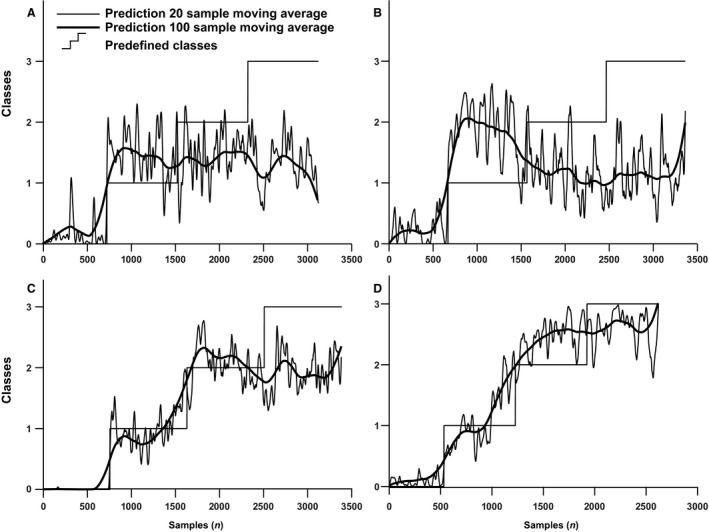
True negative and false positive results of the model. The 20 sample (thin line) and 100 sample (bold line) moving average model responses averaged over all six values for C in four subjects (panels A–D) who did not encounter presyncope within 30 min. For panels (A–C) the model did not detect any further decrease in volume state (true negatives), whereas for panel (D) the response had increased to the highest, most critical level and then reached a steady state (false positive).

The moving average of the individually predicted samples increased in classified stages of hypovolemia (Fig. 2, bottom panel) in 41 out of 46 subjects. The number of times a class was correctly classified is visualized in Fig. 3, which reveals that baseline can be distinguished from the other classes (90% correct), while the other classes show more overlap: class 1: 47% correct; class 2: 29% correct; and class 3 (60% correct). In five out of nine subjects who tolerated 30 min LBNP without development of presyncope, classification number stagnated indicating absence of progressive hypovolemia (Fig. 4).

**Figure 5:**
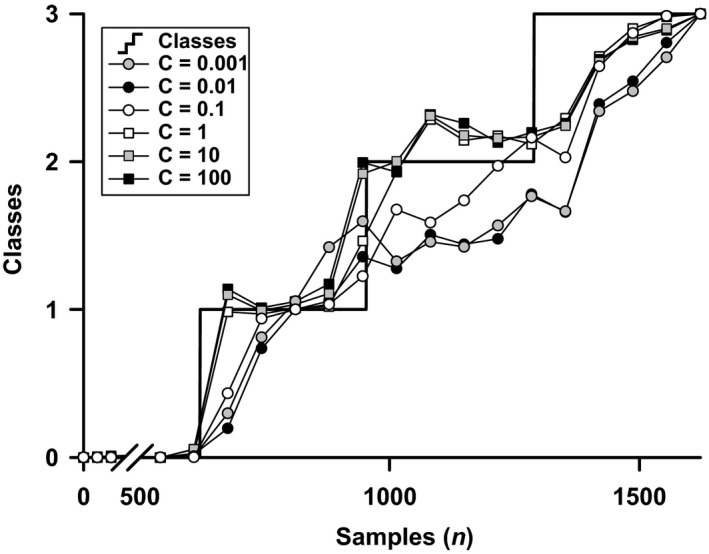
Effect of incremental value of regularization parameter C in a single subject. Artificial classes (black line, steps) to define rest (class 0) and LBNP with advancing simulated hemorrhage (class 1 through 3) and the moving averaged model classification for increasing values of regularization parameter C: C = 1e‐3; C = 1e‐2; C = 1e‐1; C = 1; C = 1e1; C = 1e2.

Incremental values for regularization variable C did not further improve model accuracy or lower MSE beyond C = 0.01 (Fig. 5 and Table 1). As expected, with further incremental values for C for classes 1 through 3 the error magnitude increased and accuracy declined. A higher value for C followed the proposed classes more strictly resulting in an artificial, stepwise quantification of hypovolemia.

**Table 1:** Model performance for all subjects experiencing presyncope

	Class 0	Class 1	Class 2	Class 3	Overall
C = 1e‐3					
Mean squared error	0.04 [0.12]	0.37 [0.70]	0.30 [0.24]	0.49 [0.89]	0.26 [0.30]
Accuracy	92 [18] %	36 [38] %	12 [9] %	56 [34] %	57 [18] %
*κ*					0.4650
C = 1e‐2					
Mean squared error	0.01 [0.10]	0.32 [0.62]	0.33 [0.23]	0.46 [0.79]	0.25 [0.35]
Accuracy	95 [16] %	34 [31] %	13 [12] %	50 [37] %	57 [18] %
*κ*					0.4816
C = 1e‐1					
Mean squared error	0.01 [0.17]	0.41 [0.54]	0.30 [0.26]	0.41 [0.73]	0.26 [0.40]
Accuracy	95 [20] %	28 [31] %	12 [15] %	46 [45] %	54 [16] %
*κ*					0.4869
C = 1					
Mean squared error	0.01 [0.20]	0.47 [0.48]	0.29 [0.20]	0.35 [0.91]	0.40 [0.36]
Accuracy	93 [27] %	26 [27] %	10 [10] %	42 [49] %	46 [19] %
*κ*					0.4047
C = 10					
Mean squared error	0.01 [0.17]	0.48 [0.61]	0.28 [0.18]	0.38 [0.92]	0.39 [0.37]
Accuracy	91 [28] %	24 [27] %	8 [8] %	38 [46] %	45 [18] %
*κ*					0.3964
C = 100					
Mean squared error	0.03 [0.36]	0.46 [0.45]	0.30 [0.23]	0.42 [0.82]	0.37 [0.43]
Accuracy	91 [36] %	21 [20] %	7 [5] %	39 [39] %	45 [15] %
*κ*					0.4456

Median [IQR] of accuracy, mean squared error per class and kappa for different values of C.

**Figure 6:**
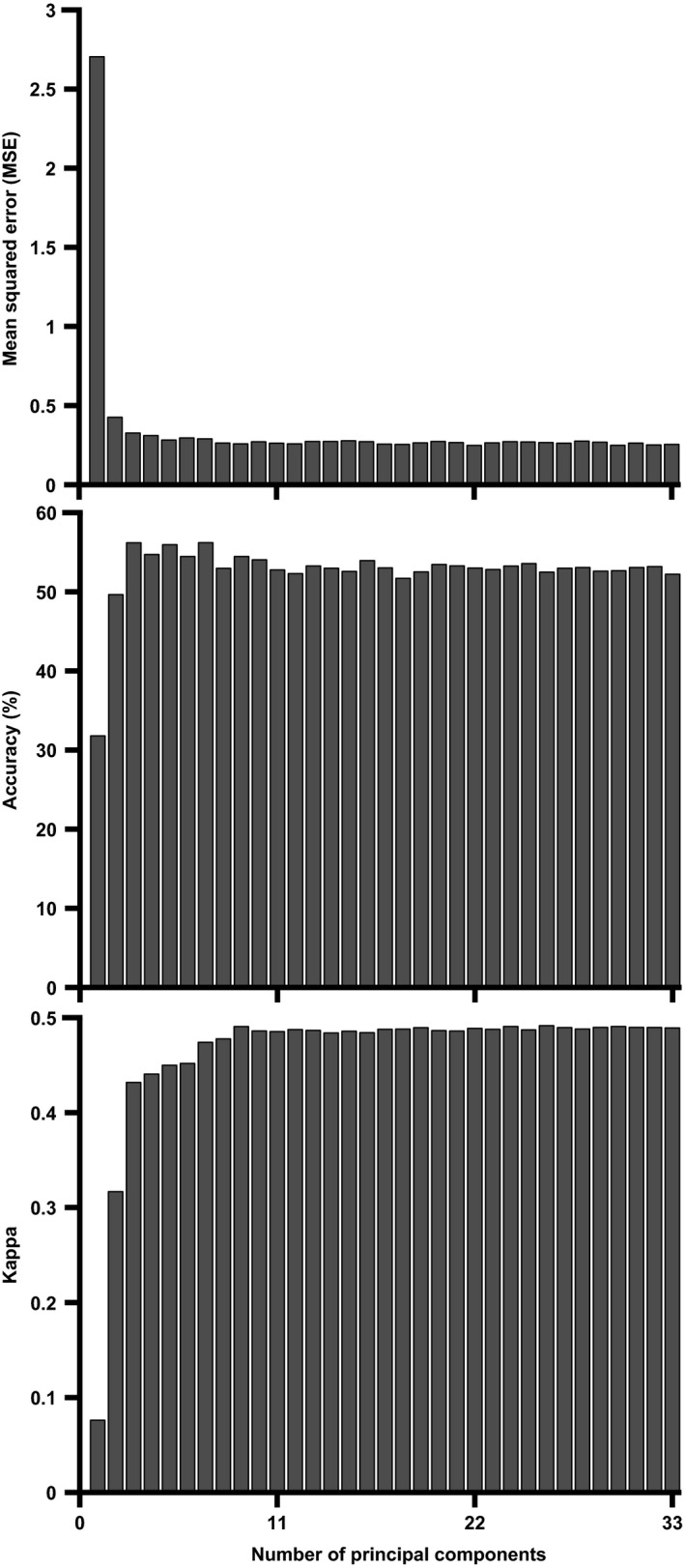
Effect of stepwise feature addition on model improvement. Model mean squared error (MSE, left) and overall accuracy (center) and kappa statistic for the model with C value: C = 1e‐2.

Accuracy and MSE improvement per added principal component revealed that the trade‐off between model improvement (as reflected by increasing values for accuracy and kappa and decreasing values of MSE) and adding more features was optimal after having added five principal components. Kappa stopped increasing after having added 10 principal components (Fig. 6).

## Discussion

The novel finding of this study is that automated feature extraction of the blood pressure waveform allows for tracking of cardiovascular events in response to ongoing depletion of the CBV up unto the point of cardiovascular collapse. We created 3 subclasses during LBNP to add additional precision to the modeled response in comparison with previous work (two classes during LBNP; van der Ster et al. 2018). The achieved accuracy implies that these subclasses are poorly defined, or may not exist from a physiological point of view respectively vary between subjects. This corresponds to distinct cardiovascular response patterns to sympathetic stimulation by LBNP in young healthy volunteers (Bronzwaer et al. 2016). The model nevertheless detected a response toward cardiovascular collapse in the majority of subjects. For most subjects who did not experience pre‐syncopal symptoms within the time frame of 30 min the progress of model classification from class 1 toward class 3 (presyncope) stagnated prematurely. This indicates that the central hypovolemia these subjects were exposed to was not progressive within that time frame. These results support that the arterial pressure waveform contains subtleties that do change with progressive hypovolemia. The pressure waveform contains information on reflection (Wilkinson et al. 2000; Dark et al. 2006), left ventricular stroke volume (Broemser and Ranke 1930; Wesseling et al. 1993) and volumetric state (Baruch et al. 2011; Convertino et al. 2011; Moulton et al. 2013). In the present study, we parametrized the arterial pressure waveform in an automated way rather than manually thus including the relevant parameters at the cost of losing a physiological meaningful interpretation of the input waveform. The principal components describe which component constitute a waveform and changes in principal components can be detected which indicates an alteration in waveform without specifying exactly what has changed. These changes in the added components appear sufficiently sensitive to classify hypovolemia and distinguish between the predefined classes. Making use of principal components exposes new sensitive information and improves identification of the degree of central hypovolemia compared to multi‐parameter monitoring (van der Ster et al. 2018).

In the present study, in contrast to the majority of LBNP studies, a single step of LBNP chamber pressure was applied until the onset of presyncope. This implies that instead of training the model to stepwise LBNP, the model recognizes labeled classes that were defined on time, meaning that there is no distinction between classes 1, 2, and 3 except time. This was performed to ensure that the algorithm was trained toward a gradual response of hypovolemia without potential induced bias set by sequential incremental levels of LBNP chamber pressure. Subjects typically suffer from a circulatory collapse following a transition from one step of LBNP to the next (Convertino et al. 2013). In this study, we made an attempt to avoid that the model would be affected and thus possibly trained by undesired rapid changes in hemodynamics. Here, the model has no knowledge about the waveform at different levels of LBNP but merely on how it changes over time. We envision a model that recognizes the subtle changes in BP waveform moving toward presyncope without being triggered by such steps. Especially when keeping generalization and the fact that these types of models are prone to overfitting in mind (notable in Fig. 5 for the higher values of C), it is undesirable to exhaustively train a model on these predefined classes which do not follow any physiological paradigm. We recognize that the adaptive behavior to environmental stress of the cardiovascular system more likely follows a nonlinear, nonclassified response pattern, and that this classification is an oversimplification of a highly complex dynamic system. This discrete approach may not do this system justice but is a prerequisite to develop a classifier. The low accuracy in class 2 indicates that there is considerable intersubject variation within this specific class. Given the results of our previous work we considered that adding a new class would allow more precision by training the model to detect more subclasses during the LBNP challenge. In the present study, this was not the case supporting that the stratification into 3 classes: rest, successfully compensatory, and close to failure was indeed sufficient. The moving averaging of the classification smooths these discrete classifications, revealing a nonlinear response in most subjects.

## Limitations and Criticism

The study population consisted of young, mostly Caucasian, healthy adults. Orthostatic differences exist for race, (Franke et al. 2004; Hinds and Stachenfeld 2010) sex (White et al. 1996; Convertino 1998; Grenon et al. 2006), and age (Wallace et al. 2010) which renders this data not directly applicable to another population such as an elderly patient population admitted to emergency care or listed for major surgery. Whether the model will track the circulatory volume state in patients is unsettled. Validation of these findings requires collection of a labeled data set with blood pressure waves from new populations during volume shifts. If the current findings hold true in a larger population of patients, it would support a wider application of this model for clinical monitoring.

Principal component analysis obscures which physiological processes determine the shape of the blood pressure waveform and thus what actually enters the model as training data, making it difficult to describe what this model is actually trained on.

## Conclusion

Progression toward syncope is tracked by a model that uses principal components of noninvasively measured continuous blood pressure waveform in healthy volunteers. Further studies should address whether the model predicts hemodynamic collapse in a real‐life setting.

## References

[phy213895-bib-0001] Abraham, W. T. , and R. W. Schrier . 1994 Body fluid volume regulation in health and disease. Adv. Intern. Med. 39:23–47. 8140955

[phy213895-bib-0002] Baruch, M. C. , D. E. Warburton , S. S. Bredin , A. Cote , D. W. Gerdt , and C. M. Adkins . 2011 Pulse Decomposition Analysis of the digital arterial pulse during hemorrhage simulation. Nonlinear Biomed. Phys. 5:1. 21226911 10.1186/1753-4631-5-1 PMC3025935

[phy213895-bib-0003] Bennis, F. C. , B. J. van der Ster , J. J. van Lieshout , P. Andriessen , and T. Delhaas . 2017 A machine‐learning based analysis for the recognition of progressive central hypovolemia. Physiol. Meas. 38:1791–1801. 28671554 10.1088/1361-6579/aa7d3d

[phy213895-bib-0004] Bishop, M. C. 2006 Pattern recognition and machine learning. Springer Science+Business Medica, LLC, Cambridge.

[phy213895-bib-0005] Broemser, P. , and O. F. Ranke . 1930 Uber die Messung des Schlagvolumens des Herzens auf unblutigem Weg. Z. Biol. 90:467–507.

[phy213895-bib-0006] Bronzwaer, A. S. , D. M. Ouweneel , W. J. Stok , B. E. Westerhof , and J. J. van Lieshout . 2015 Arterial pressure variation as a biomarker of preload dependency in spontaneously breathing subjects – a proof of principle. PLoS One 10:e0137364. 26335939 10.1371/journal.pone.0137364 PMC4559442

[phy213895-bib-0007] Bronzwaer, A.‐S. , J. Verbree , W. J. Stok , M. A. van Buchem , M. J. Daemen , M. J. van Osch , et al. 2016 Cardiovascular response patterns to sympathetic stimulation by central hypovolemia. Front. Physiol. 7:235. 27378944 10.3389/fphys.2016.00235 PMC4913112

[phy213895-bib-0008] Bronzwaer, A. G. , J. Verbree , W. J. Stok , M. J. Daemen , M. A. van Buchem , M. J. van Osch , et al. 2017 The cerebrovascular response to lower‐body negative pressure vs. head‐up tilt. J. Appl. Physiol. (1985) 122:877–883. 28082333 10.1152/japplphysiol.00797.2016

[phy213895-bib-0009] Chang, C. , and C. Lin . 2011 LIBSVM: a library for support vector machines. ACM Trans. Intel. Syst. Technol. 2:1–27.

[phy213895-bib-0010] Cohen, J. 1960 A coefficient of agreement for nominal scales. Educ. Psychol. Measur. 20:37–46.

[phy213895-bib-0011] Convertino, V. A. 1998 Gender differences in autonomic functions associated with blood pressure regulation. Am. J. Physiol. 275:R1909–R1920. 9843880 10.1152/ajpregu.1998.275.6.R1909

[phy213895-bib-0012] Convertino, V. A. , S. L. Moulton , G. Z. Grudic , C. A. Rickards , C. Hinojosa‐Laborde , R. T. Gerhardt , et al. 2011 Use of advanced machine‐learning techniques for noninvasive monitoring of hemorrhage. J. Trauma 71:S25–S32. 21795890 10.1097/TA.0b013e3182211601

[phy213895-bib-0013] Convertino, V. A. , G. Grudic , J. Mulligan , and S. Moulton . 2013 Estimation of individual‐specific progression to impending cardiovascular instability using arterial waveforms. J. Appl. Physiol. (1985) 115:1196–1202. 23928113 10.1152/japplphysiol.00668.2013

[phy213895-bib-0014] Dark, P. , R. Little , M. Nirmalan , and J. Purdy . 2006 Systemic arterial pressure wave reflections during acute hemorrhage. Crit. Care Med. 34:1497–1505. 16540954 10.1097/01.CCM.0000215451.26971.89

[phy213895-bib-0015] Franke, W. D. , K. Lee , D. B. Buchanan , and J. P. Hernandez . 2004 Blacks and whites differ in responses, but not tolerance, to orthostatic stress. Clin. Auton. Res. 14:19–25. 15045596 10.1007/s10286-004-0155-5

[phy213895-bib-0016] Fu, Q. , S. Witkowski , and B. D. Levine . 2004 Vasoconstrictor reserve and sympathetic neural control of orthostasis. Circulation 110:2931–2937. 15505093 10.1161/01.CIR.0000146384.91715.B5

[phy213895-bib-0017] Godfrey, G. E. , S. W. Dubrey , and J. M. Handy . 2014 A prospective observational study of stroke volume responsiveness to a passive leg raise manoeuvre in healthy non‐starved volunteers as assessed by transthoracic echocardiography. Anaesthesia 69:306–313. 24641636 10.1111/anae.12560

[phy213895-bib-0018] Goswami, N. , E. Grasser , A. Roessler , D. Schneditz , and H. Hinghofer‐Szalkay . 2009 The cardiovascular response to lower body negative pressure in humans depends on seal location. Physiol. Res. 58:311–318. 18637716 10.33549/physiolres.931431

[phy213895-bib-0019] Grenon, S. M. , X. Xiao , S. Hurwitz , N. Sheynberg , C. Kim , E. W. Seely , et al. 2006 Why is orthostatic tolerance lower in women than in men? Renal and cardiovascular responses to simulated microgravity and the role of midodrine. J. Investig. Med. 54:180–190. 10.2310/6650.2006.05064 17152857

[phy213895-bib-0020] Harms, M. P. M. , J. J. van Lieshout , M. Jenstrup , F. Pott , and N. H. Secher . 2003 Postural effects on cardiac output and mixed venous oxygen saturation in humans. Exp. Physiol. 88:611–616. 12955161 10.1113/eph8802580

[phy213895-bib-0021] Hinds, K. , and N. S. Stachenfeld . 2010 Greater orthostatic tolerance in young black compared with white women. Hypertension 56:75–81. 20458005 10.1161/HYPERTENSIONAHA.110.150011 PMC2909588

[phy213895-bib-0022] Johnson, B. D. , N. van Helmond , T. B. Curry , C. M. van Buskirk , V. A. Convertino , and M. J. Joyner . 2014 Reductions in central venous pressure by lower body negative pressure or blood loss elicit similar hemodynamic responses. J. Appl. Physiol. 117:131–141. 24876357 10.1152/japplphysiol.00070.2014 PMC4459917

[phy213895-bib-0023] Kalantari, K. , J. N. Chang , C. Ronco , and M. H. Rosner . 2013 Assessment of intravascular volume status and volume responsiveness in critically ill patients. Kidney Int. 83:1017–1028. 23302716 10.1038/ki.2012.424

[phy213895-bib-0024] Landis, J. R. , and G. G. Koch . 1977 The measurement of observer agreement for categorical data. Biometrics 33:159–174. 843571

[phy213895-bib-0025] Marik, P. E. , R. Cavallazzi , T. Vasu , and A. Hirani . 2009 Dynamic changes in arterial waveform derived variables and fluid responsiveness in mechanically ventilated patients: a systematic review of the literature. Crit. Care Med. 37:2642–2647. 19602972 10.1097/CCM.0b013e3181a590da

[phy213895-bib-0026] Marik, P. , X. Monnet , and J. L. Teboul . 2011 Hemodynamic parameters to guide fluid therapy. Ann. Intensive Care 1:1–9. 21906322 10.1186/2110-5820-1-1 PMC3159904

[phy213895-bib-0027] Martina, J. R. , B. E. Westerhof , J. van Goudoever , E. M. de Beaumont , J. Truijen , Y. S. Kim , et al. 2012 Noninvasive continuous arterial blood pressure monitoring with Nexfin(R). Anesthesiology 116:1092–1103. 22415387 10.1097/ALN.0b013e31824f94ed

[phy213895-bib-0028] McMichael, J. 1944 Clinical aspects of shock. J. Am. Med. Assoc. 124:275–281.

[phy213895-bib-0029] Moulton, S. L. , J. Mulligan , G. Z. Grudic , and V. A. Convertino . 2013 Running on empty? The compensatory reserve index. J. Trauma Acute Care Surg. 75:1053–1059. 24256681 10.1097/TA.0b013e3182aa811a

[phy213895-bib-0030] Pearson, K. 1901 LIII. On lines and planes of closest fit to systems of points in space. Philos. Mag. 2:559–572.

[phy213895-bib-0031] Rickards, C. A. , B. D. Johnson , R. E. Harvey , V. A. Convertino , M. J. Joyner , and J. N. Barnes . 2015 Cerebral blood velocity regulation during progressive blood loss compared to lower body negative pressure in humans. J. Appl. Physiol. (1985) 119:677–685. 26139213 10.1152/japplphysiol.00127.2015 PMC4747890

[phy213895-bib-0032] Sander‐Jensen, K. , N. H. Secher , P. Bie , J. Warberg , and T. W. Schwartz . 1986 Vagal slowing of the heart during haemorrhage: observations from 20 consecutive hypotensive patients. BMJ 292:364–366. 3080172 10.1136/bmj.292.6517.364 PMC1339346

[phy213895-bib-0033] Schiller, A. M. , J. T. Howard , and V. A. Convertino . 2017 The physiology of blood loss and shock: new insights from a human laboratory model of hemorrhage. Exp. Biol. Med. (Maywood) 242:874–883. 28346013 10.1177/1535370217694099 PMC5407541

[phy213895-bib-0034] Schondorf, R. , and W. Wieling . 2000 Vasoconstrictor reserve in neurally mediated syncope. Clin. Auton. Res. 10:53–55. 10823335 10.1007/BF02279891

[phy213895-bib-0035] Schrier, R. W. 1990 Body fluid volume regulation in health and disease: a unifying hypothesis. Ann. Intern. Med. 113:155–159. 2193561 10.7326/0003-4819-113-2-155

[phy213895-bib-0036] Secher, N. H. , and J. J. van Lieshout . 2005 Normovolaemia defined by central blood volume and venous oxygen saturation. Clin. Exp. Pharmacol. Physiol. 32:901–910. 16405445 10.1111/j.1440-1681.2005.04283.x

[phy213895-bib-0037] Secher, N. H. , and van Lieshout J. J. 2016 Hypovolemic shock Pp. 222–231 in Hahn R. G., ed. Clinical fluid therapy in the perioperative setting. 2nd ed. Cambridge University Press, Cambridge.

[phy213895-bib-0038] Shao, Z. , M. J. Er , and N. Wang . 2016 An Efficient Leave‐One‐Out Cross‐Validation‐Based Extreme Learning Machine (ELOO‐ELM) with minimal user intervention. IEEE Trans. Cybern. 46:1939–1951. 26259254 10.1109/TCYB.2015.2458177

[phy213895-bib-0039] van der Ster, B. J. P. , F. C. Bennis , T. Delhaas , B. E. Westerhof , W. J. Stok , and J. J. van Lieshout . 2018 Support vector machine based monitoring of cardio‐cerebrovascular reserve during simulated hemorrhage. Front. Physiol. 8:1057. 29354062 10.3389/fphys.2017.01057 PMC5761201

[phy213895-bib-0040] Wallace, J. P. , G. T. Trail , and W. D. Franke . 2010 LBNP tolerance analyzed retrospectively using a structural equation model. Aviat. Space Environ. Med. 81:363–368. 20377138 10.3357/asem.2563.2010

[phy213895-bib-0041] Wesseling, K. H. , J. R. C. Jansen , J. J. Settels , and J. J. Schreuder . 1993 Computation of aortic flow from pressure in humans using a nonlinear, three‐element model. J. Appl. Physiol. 74:2566–2573. 8335593 10.1152/jappl.1993.74.5.2566

[phy213895-bib-0042] White, D. D. , R. W. Gotshall , and A. Tucker . 1996 Women have lower tolerance to lower body negative pressure than men. J. Appl. Physiol. (1985) 80:1138–1143. 8926238 10.1152/jappl.1996.80.4.1138

[phy213895-bib-0043] Wiggers, C. J. 1950 Physiology of shock. The Commonwealth Fund, New York.

[phy213895-bib-0044] Wilkinson, I. B. , H. Maccallum , L. Flint , J. R. Cockcroft , D. E. Newby , and D. J. Webb . 2000 The influence of heart rate on augmentation index and central arterial pressure in humans. J. Physiol. 525:263–270. 10811742 10.1111/j.1469-7793.2000.t01-1-00263.x PMC2269933

[phy213895-bib-0045] Zhang, G. , K. L. Ryan , C. A. Rickards , V. A. Convertino , and R. Mukkamala . 2012 Early detection of hemorrhage via central pulse pressure derived from a non‐invasive peripheral arterial blood pressure waveform. Conf. Proc. IEEE Eng. Med. Biol. Soc. 2012:3116–3119. 23366585 10.1109/EMBC.2012.6346624

